# Relaxing Retinotomy in Recurrent and Refractory Full-Thickness Macular Holes: The State of the Art

**DOI:** 10.3390/life13091844

**Published:** 2023-08-31

**Authors:** Luca Ventre, Erik Mus, Fabio Maradei, Roberto Imparato, Giulia Pintore, Guglielmo Parisi, Paola Marolo, Michele Reibaldi

**Affiliations:** 1 Department of Ophthalmology, Beauregard Hospital, Azienda USL della Valle d’Aosta, Via L. Vaccari 5, 11100 Aosta, Italy; 2 Department of Ophthalmology, University of Turin, Via Cherasco 23, 10126 Turin, Italy

**Keywords:** full-thickness macular hole, recurrent macular hole, persistent macular hole, refractory macular hole, relaxing retinotomy, surgery repair, vitrectomy, macular holes

## Abstract

The prevailing standard of care for primary repair of full-thickness macular holes (FTMHs) is pars plana vitrectomy with internal limiting membrane (ILM) peeling and gas tamponade, as it gives a high closure rate of roughly 90%. On the other hand, the surgical management of recurrent and refractory FTMHs represents, so far, a demanding and debated subject in vitreoretinal surgery since various approaches have been proposed, with no consensus concerning both adequate selection criteria and the best surgical approach. In addition, the existence of multiple case series/interventional studies showing comparable results and the lack of studies with a direct comparison of multiple surgical techniques may lead to uncertainty. We present an organized overview of relaxing retinotomy technique, a surgical approach available nowadays for the secondary repair of recurrent and refractory FTMHs. Besides the history and the description of the various techniques to perform relaxing retinotomies, we underline the results and the evidence available to promote the use of this surgical approach.

## 1. Introduction

In the overall population older than 60 years, idiopathic full-thickness macular holes (FTMHs) present a 2.2–3:1 female preponderance and reveal a prevalence of approximately 0.3–5%, growing with age [1,2,3]. The current gold standard for the primary repair of FTMHs is pars plana vitrectomy (PPV) with internal limiting membrane (ILM) peeling and gas tamponade, leading to FTMH closure in 80–100% of overall cases [4,5,6,7,8]. Even though there is a positive success rate, primary repair may be unsuccessful or a late reopening of the full-thickness macular hole may occur [9].

When a FTMH does not close after the primary surgery, it is described as “persistent or refractory”, while if a reopening is observed at least 4 weeks after initial successful closure it is defined as “recurrent” [10]. Planning a secondary surgery, vitreoretinal surgeons need to take into account some limitations associated with recurrent and refractory FTMHs including decreased quantity or lack of ILM to peel, nonfeasibility of ILM inverted flap, and lower success percentage of repeated surgeries [11]; nevertheless, a second surgical repair seems to be justified by the functional and anatomical potential achievable results [12,13]. As a matter of fact, in more than 75% of patients a secondary closure may be obtained [12], and closure of recurrent or refractory FTMHs may result in a remarkable visual improvement in the bulk of patients [12,14]. In addition, the natural history of an open FTMH tends to enlargement, causing progressive atrophy and loss of vision [15], while multiple surgeries can more rarely lead to deterioration of vision [14].

For the management of recurrent and refractory FTMHs, multiple surgical techniques may be performed; however, the scientific literature is restricted for some of them because they were newly introduced in the surgery field [9]. Consequently, a common consensus concerning the most efficient technique for secondary repair [16] of FTMHs is not yet achieved, in contrast to the primary surgery. Numerous techniques have been suggested to deal with FTMHs that do not definitively close after surgery [9], such as autologous platelet-rich plasma (aPRP) [17], revisional pars plana vitrectomy without or with internal limiting membrane peeling enlargement [18], subretinal fluid (SRF) injection [19], relaxing retinotomies, ambulatory fluid—gas exchange [20] without or with retinal LP (laser photocoagulation), retinal massage [21], microdraining [15,22], macular buckling [23], autologous neurosensory retinal free flap [24], human amniotic membrane (hAM) graft [9,25,26], ILM free flap with or without autologous blood [27] and lens capsular flap (LCF) [28,29].

Concerning relaxing retinotomies [30,31,32,33,34,35], in different case series both the surgical technique and the position of retinotomies vary considerably. We present a comprehensive and structured overview of the relaxing retinotomy technique, a surgical approach available nowadays for the secondary repair of recurrent and refractory FTMHs.

Besides the history and the description of the various techniques to perform relaxing retinotomies, we underline the results and the evidence available to promote the use of this surgical approach.

## 2. Material and Methods

We reviewed the current literature concerning relaxing retinotomies for recurrent and refractory full-thickness macular holes utilizing the PubMed and Embase databases until November 2022 and the following keywords: “relaxing retinotomy”; “macular hole”; “refractory full-thickness macular holes”; “persistent full-thickness macular holes”; “relaxing retinotomies”; and “recurrent full-thickness macular hole” in various combinations.

We analyzed every remarkable publication written in English. We included all the available studies (case series, case reports, original articles) and authoritative reviews on this topic. Abstract-only articles or articles in non-English languages whose translations were not provided were excluded.

## 3. Surgical Techniques

Utilizing the keywords written above, 67 articles were found, 8 of which presented the use of relaxing retinotomy for the repair of recurrent and refractory FTMHs. Table 1 shows an overview of the most important studies discussed in this review.

In 2006, Charles and coworkers [36] presented firstly the use of temporal arcuate partial thickness retinotomy for full-thickness macular holes that failed primary surgery at the Macula Society 29th annual meeting in California [31].

Michalewska et al. [37] and Alpatov and coworkers [38] illustrated a surgical approach where the edges of the hole were mechanically pushed together with forceps with the objective of reducing the dimension of the hole [31]. There was poor functional success, even though anatomical results were slightly improved [31].

Smiddy [39] performed short incisions at the border of the hole in order to promote retinal gliosis. However, anatomical and functional results were not made available [31].

**Figure 1:**
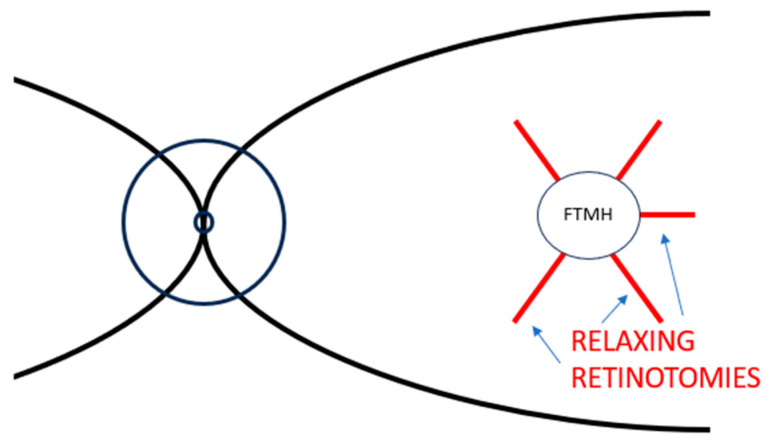
Stylized representation of a retinal posterior pole with FTMH and relaxing retinotomies suggested by Reis and coworkers: five full-thickness perifoveal radial retinal incisions. Abbreviation: FTMH, full-thickness macular holes.

In 2012, Reis et al. [31] carried out full thickness radial retinal incisions on seven eyes with FTMHs that remained unclosed after initial pars plana vitrectomy with ILM peeling. Five full-thickness perifoveal radial incisions utilizing a 25-gauge needle or a barbed microvitreoretinal blade were created starting one macular hole diameter away from its margins and extended centripetally until the edges of the hole, avoiding the area of the papillomacular bundle (Figure 1). At the end, tamponade with 0.8 mL of 100% sulfur hexafluoride (SF_6_) was executed and face-down positioning was recommended for seven days.

Anatomical closure was reached in all patients with no loss of best corrected visual acuity (BCVA) in every patient, and mean visual acuity improved by 5.6 lines. Surgeries were uneventful, excluding low perifoveal bleeding during the operation; in addition, there were no other postoperative complications.

These incisions are believed to produce a relaxing effect on the perifoveal tissue and to stimulate retinal gliosis, thus promoting the closure of the macular hole. They can possibly lead to upregulation of gliosis or foster the supposed centripetal movement of retinal tissue towards the fovea, as referred by Hillenkamp and coworkers. [11] Reis et al. [31] think that if a deep retinal perifoveal incision is made right to the RPE (retinal pigment epithelium), it will induce a stress response and the RPE will react with cell migration and proliferation. Thus, macular hole closure would be accelerated by the stimulatory effect on retinal gliosis and on the development of the glial scar.

A resembling surgical approach of six partial thickness incisions made with a MVR blade, 1‒2 mm long in a radial pattern and avoiding the papillomacular bundle, resulted in 82% closure (22 out of 27) of full-thickness macular holes that had failed to close following primary repair, with mean hole size of 488 µm [15].

In 2013, Charles and coworkers [30] reported the use of a full thickness arcuate relaxing retinotomy temporal to a macular hole in a series of six phakic eyes of six patients with large FTMHs (mean diameter 658 ± 180 µm) that had failed to close following primary repair.

Primary surgery including 25G pars plana vitrectomy, removal of posterior vitreous cortex from the retinal surface, internal limiting membrane peeling and fluid-gas exchange had been previously performed on all patients without success. During primary or secondary surgery, epiretinal staining (indocyanine green) was not applied.

**Figure 2:**
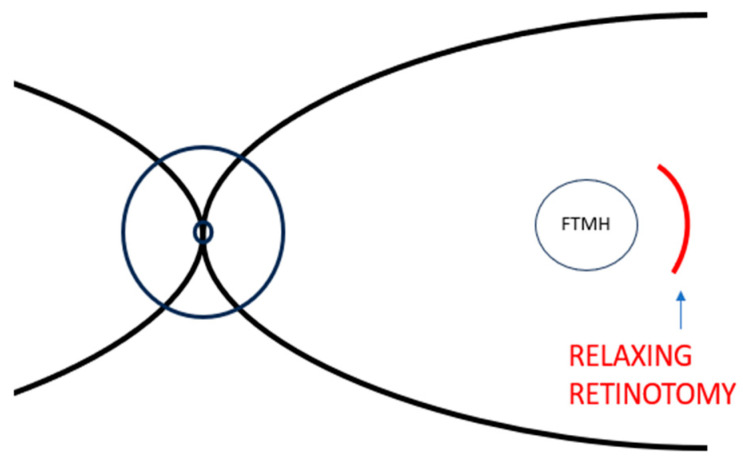
Stylized representation of a retinal posterior pole with FTMH and relaxing retinotomy proposed by Charles and coworkers: full thickness arcuate (semicircular) retinotomy temporal to a macular hole. Abbreviation: FTMH, full-thickness macular holes.

Charles et al. [30] performed the arcuate (semicircular) retinotomy during 25G PPV at 500–700 μm from the temporal margin of the FTMH, utilizing disposable 25G curved scissors (Figure 2). The arc length of the retinotomy was centered 90° on the temporal horizontal meridian, with the purpose of following the course of the nerve fibers. After realizing the arcuate incision, fluid‒air exchange was made, followed by internal drainage through the hole of any subretinal fluid. A simultaneous inward dislocation of the temporal retinal bridge between the margin of the FTMH and the retinotomy was executed using a 25G soft tip cannula. This resulted in horizontal shortening and vertical elongation of the FTMH and a widening of the temporal retinotomy site. Eventually air‒gas exchange with 18% C_3_F_8_ (perfluoropropane) or 25% SF_6_ was completed and patients were given instruction to hold a face-down position in the postoperative period for one week.

The idea that lies beneath arcuate temporal retinotomy is to give additional retinal compliance not achievable with ILM peeling alone, favoring dislocation of the temporal retinal tissue and closure of FTMHs [30]. Charles et al. observed that the association of arcuate retinotomy and ILM peeling increments retinal compliance and promotes closure of wide FTMHs that might otherwise remain open with conventional surgical procedures.

Using this technique, five (83%) of six eyes achieved FTMH closure and three (50%) evidenced an improvement in visual acuity [30]. The study evidenced that no patient experienced a loss of visual acuity from baseline or reported additional scotoma in the nasal visual field after the temporal arcuate retinotomy approach. A formal Humphrey visual field (HVF) exam was executed with success on two patients. The first patient’s visual field study revealed no scotoma but was regarded as untrustworthy owing to poor fixation, while the second patient’s visual field study showed a nasal scotoma that was clinically asymptomatic in the area corresponding to the retinotomy.

Nevertheless, although OCT postoperative evaluation of the retinotomy sites evidenced closed retinotomies in all cases, some patients showed RPE defects and retinal thinning at the retinotomy site [30].

According to follow-up OCT scans, the authors considered nasal scotoma to be secondary to RPE scissors trauma, localized retinal atrophy, or secondary to transection of the axons of the ganglion cells.

It is well acknowledged that retinal ganglion cell axons temporal to the fovea and adjacent to the horizontal raphe run below and above the macula and the papillomacular bundle while they enter the optic nerve [30]. Hence, their configuration as they leave the horizontal raphe is oblique, near vertical [30]. This anatomic knowledge allows for the realization of an arcuate retinotomy that follows the course of the retina ganglion cell axons, thus preventing transection of most nerve fibers and, as a consequence, the development of a nasal scotoma [30].

In 2017, Tsipursky was the first to employ endocautery and a soft tip cannula to realize a paracentral retinotomy in six patients that previously had undergone at least one unsuccessful PPV for large FTMHs [35]. Owing to this procedure, 83% of cases reached anatomical closure and mean visual acuity ameliorated from 1.28 logMAR at baseline to 0.89 logMAR after the surgery. At follow-up controls, none of the patients experienced deterioration of vision, without evidence of retinal nerve fiber layer (RNFL) loss on OCT or optic neuropathy [33]. Patients tolerated well paracentral scotomas detected in their visual field tests.

Karacorlu et al. [34] presented a patient who showed a macular tractional rhegmatogenous retinal detachment and a wide FTMH after PPV for proliferative diabetic retinopathy. In fact, secondary FTMH formation may develop after PPV for proliferative diabetic retinopathy or retinal detachment, even though idiopathic FTMH is the most frequent presentation [40].

After that, ILM peeling failed to close the FTMH; inferior and superior posterior arcuate 120° relaxing retinotomies were executed with scissors under perfluorocarbon liquid, near the inferotemporal and superotemporal vascular arcades, achieving FTMH closure but restricted functional improvement [34]. During retinotomy, in order to control hemorrhage, the infusion pressure was augmented and endodiathermy was used to cauterize blood vessels.

Eventually, laser photocoagulation was performed along the posterior arcuate relaxing retinotomies. At the end of the surgery, silicone oil (SO) was used as internal tamponade. No postoperative or intraoperative complications occurred. The authors opted for double relaxing retinotomy in order to decrease traction, allowing symmetrical reapproximation of FTMH margins and retinal attachment [34].

Anatomical and functional results related to relaxing nasal retinotomy technique for closure of recurrent FTMH were illustrated in Knight’s study [33]. In that case report, a female patient aged 69 with juxtafoveal telangiectasia and a recurrent macular hole (425 µm) after primary PPV and ILM peeling underwent a second standard 3-port 25G PPV. Superonasal to the macula, a single relaxing retinotomy was realized utilizing diathermy followed by gentle suction with a soft tip cannula of the retinotomy site [33].

After the procedure was accomplished, the FTMH seemed smaller. Thus, the intraoperative OCT (iOCT) measured the FTMH immediately after the nasal retinotomy and the dimension of the macular hole had reduced to 219 µm. Then, a complete fluid‒air exchange (FAX) was carried out utilizing a soft tip cannula, followed by injection of C_3_F_8_ and 7-day face-down postoperative posturing.

At the 1-week, 1-month, and 6-month postoperative follow-up controls, both the FTMH and the nasal retinotomy site were completely closed. Follow-up related some visual change with BCVA improving from 20/200 to 20/100 after secondary repair. No postoperative complications occurred.

In 2021, the study of Tsipursky [32] extended on his previous work [35] and on Knight’s [33] case in order to illustrate the usefulness of nasal relaxing retinotomy in a more numerous cohort of patients with refractory FTMHs and a more extended follow-up. It was a multicenter, retrospective, interventional study of patients with refractory FTMHs who previously had undergone one or more pars plana vitrectomy with peeling of the internal limiting membrane.

**Figure 3:**
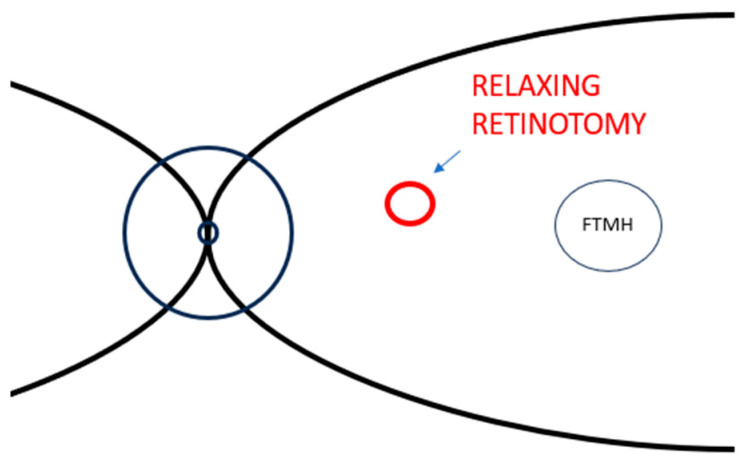
Stylized representation of a retinal posterior pole with FTMH and relaxing retinotomy presented by Tsipursky and coworkers: paracentral nasal relaxing retinotomy. Abbreviation: FTMH, full-thickness macular holes.

It enlisted 13 patients, with an average age of 65 years (from 49 to 90 years old), that were all subjected to 25G transconjunctival PPV. First, intravitreal indocyanine green-stained residual ILM and, if present, a wider circumferential ILM peeling was carried out. Then, a gentle endodiathermy was performed with a 25G tapered tip to realize a small retinotomy (Figure 3), displayed by a tiny area of whitening on the retina, at the midpoint between the optic disc and the fovea [32]. Next, gentle suction with a soft tip cannula was performed at the relaxing parafoveal nasal retinotomy site; finally, this was followed by FAX and further suction in order to drain completely the FTMH. At the end of surgery, SF_6_ or C_3_F_8_ was injected, and a face-down position in the postoperative period was recommended after the procedure for a period of 1 week to 10 days.

In this study [32], 12 of 13 eyes (92.3%) attained anatomical closure after a median follow-up of one year. Analyzing these 12 eyes that achieved closure, 10 eyes showed macular holes greater than 400 µm of diameter, and partial or complete restoration of the subfoveal ellipsoid zone was observed in 8 eyes. This surgical approach was unsuccessful in resolving one chronic full-thickness macular hole that had undergone three earlier unsuccessful repair surgeries; nevertheless, this patient improved postoperative visual acuity. According to the postoperative OCT exams, five out of eight eyes maintained open the retinotomy site. However, RNFL thickness and open retinotomy sites remained stable during 12-month median duration of follow-up, without postoperative presence of SRF or enlargement of the open retinotomy [32].

Curiously, four of five eyes with chronic FTMHs (persisted for 6 months or more) reached anatomical closure with parafoveal nasal relaxing retinotomy. In these eyes, mean visual acuity (±standard error) increased considerably from 1.63 ± 0.32 logMAR to 0.75 ± 0.19 logMAR. These achievements entail that the chronicity of a FTMH should not preclude any surgical attempts of resolution with PPV and relaxing nasal retinotomy [32].

On the whole, 9 out of 13 patients vision in the course of postoperative controls, and mean visual acuity ameliorated considerably from 1.20 ± 0.15 logMAR to 0.84 ± 0.11 logMAR.

Postoperative Humphrey 24-2 and/or 10-2 visual field did not reveal identifiable defect in three out of eleven eyes, whereas in the remaining eight eyes, small paracentral scotomas and an enlarged central scotoma were noted.

Globally, patients countenanced paracentral scotomas in a good manner. They preferred an enhanced vision with postoperative paracentral scotoma compared to central scotoma resulting from FTMH [32].

Tsipursky et al. observed that the most foreshortened area among eyes with persistent FTMHs was frequently the nasal macula and they believed that the temporal relaxing retinotomy may imply more risk of retinal detachment than parafoveal nasal relaxing retinotomy; for this reason, they practiced the aforementioned surgical approach [32].

Reducing tangential traction by the adjacent retina on the macular hole [16,30,33,34] is the suggested mechanism by means of which relaxing retinotomies facilitate FTMH closure, even though augmented glial cell proliferation may play a part [31].

In Tsipursky’s study [32], a gentle suction utilizing a soft tip cannula at the parafoveal nasal retinotomy site followed retinotomy, in order to drain completely the FTMH. During the surgery, Tsipursky noticed that suction instantly permitted the borders of the FTMH to draw closer and shrank the diameter of the macular hole, advising that the abovementioned method might ulteriorly facilitate FTMH closure by additional relaxing the adjacent retinal tissue [32]. Moreover, tissue manipulation and fluid aspiration may help by lysing adhesions between the RPE underlying the FTMH and its margins that otherwise preclude macular hole closure [32].

Even though Tsipursky et al. are supporters of the primary surgery of large FTMHs of the ILM flap or scaffolding technique, the achievements of this case series indicate that relaxing parafoveal nasal retinotomy may promote functional improvement and long-term anatomical closure in patients with recurrent or persistent FTMHs [32].

## 4. Discussion

The encouraging results of the various available surgical techniques seem to support the decision to reoperate recurrent and refractory full-thickness macular holes; nevertheless, a direct comparison between the different surgical procedures is hard and challenging due to the scarcity of large prospective comparative studies and/or randomized controlled trials (RCTs) [9]. An evidence-based consensus of the best surgical technique has not been proved yet; consequently, a consensus approach for treating recurrent and refractory full-thickness macular holes is yet to come.

The underlying reason for employing relaxing retinotomy for the treatment of recurrent and refractory macular holes consists in decreasing the tangential traction applied by the adjacent retina on the FTMH and increasing the retinal compliance [16,30,33,34], consequently favoring the hole closure [30]. Furthermore, the retinal gliosis may be stimulated by the surgical injury connected to the retinotomy, playing a role in the hole closure [31].

Advantages of the relaxing retinotomy technique for recurrent and refractory FTMH repair encompass preservation of central visual acuity, prompt decreasing of FTMH diameter visualized with intraoperative OCT, its belonging to surgical technique usually performed by posterior segment surgeons and feasibility in previously vitrectomized eyes. Moreover, we believe that in comparison to other proposed techniques (such as human amniotic membrane graft, autologous neurosensory retinal free flap or lens capsular flap…), in the relaxing retinotomy technique there is not a manipulation of the perifoveal tissue, thus avoiding damage to perilesional photoreceptors.

In contrast, relaxing retinotomy is a “destructive” surgical approach that sacrifices healthy tissue to foster FTMH closure. Performing retinotomies may possibly cause traumatic injury to the underlying RPE owing to the inability to evaluate exactly the depth of incision under the retina that is completely adherent at the time of penetration.

In spite of the published high closure rate, the relaxing retinotomy technique involves a risk of inducing paracentral scotomas. Nevertheless, Tsipursky’s study [32] found that the appearance of small nasal and temporal scotomas generally weighed less than the functional visual acuity achievements obtained during postoperative controls by most patients.

Next, it must be considered that the previous analyzed studies did not include patients with remarkable macular pathologies (e.g., significant diabetic retinopathy, retinal vein occlusions or advanced age-related macular degeneration). We agree with Tsipursky and coworkers [32], who suggest that these patients should not undergo relaxing retinotomy, as well as patients with severe glaucoma who potentially face postoperative RNFL thinning [32].

Furthermore, Frisina et al. [41] compared different surgical approaches for recurrent or refractory full-thickness macular holes repair with regard to visual gain and closure rate. They concluded that the relaxing retinotomy technique and its intrinsic complexity may not be justified in comparison to presently available easier and more effective surgical alternatives [9]. Nevertheless, concerning the relaxing retinotomy technique, Frisina’s publication takes into account only two studies [30,31], without considering the newer developments [32,33,35] in this procedure. Recent studies give evidence that this technique is becoming easier to perform; indeed Tsipursky illustrates a simpler, novel and safer option that may be easily adopted in standard surgery. Moreover, if the posterior pole relaxing retinotomy achieves good success in highly myopic patients with recurrent macular hole retinal detachment [42], it is possible to extrapolate that relaxing retinotomies could be useful in recurrent and refractory full-thickness macular holes.

## 5. Conclusions

The available literature on relaxing retinotomies for recurrent and refractory FTMHs indicates that this surgical practice reaches a high percentage of FTMH anatomical closure with different grades of functional improvement. In conclusion, relaxing retinotomies may be a successful procedure to facilitate anatomical closure of macular holes and enhance visual acuity in eyes with recurrent and refractory FTMHs.

However, the currently available studies show a number of shortcomings, including small sample size, retrospective design, heterogeneity of surgical steps, sample, and methods. These studies enlist a restricted number of patients and visual field tests are not performed in all of them, impeding the capacity to assess the existence and location of subsequent scotomas.

It is essential to carry out further studies based on a large sample size, homogeneous protocol, and standardized surgical procedures to better evaluate the anatomical and functional success rate of the relaxing retinotomy, the best location of the incision, its possible complications, and the patient population for which this procedure is most applicable. Even more important, future studies are necessary to comprehensively compare the multiple surgical techniques available for the secondary surgery of recurrent and refractory FTMHs regarding anatomical and functional outcomes.

## Figures and Tables

**Table 1:** Major studies on relaxing retinotomy for the repair of recurrent and refractory FTMHs.

Study ^a^	Year	Study Type	Type of Relaxing Retinotomy	Number of Patients	Instruments	Intraoperative or Postoperative Complications	Tamponade	Results/Conclusions
Reis et al. [31] (PMID: 23071465)	2012	Case series, monocentric, retrospective, interventional study	Five full-thickness perifoveal radial retinal incisions, starting one macular hole diameter away from its margins and extended centripetally until the edges of the FTMH	7	25-gauge needle or a barbed micro-vitreoretinal blade	Low perifoveal bleeding during the operation	SF_6_	Anatomical closure achieved in all patients and mean VA improved by 5.6 lines
Charles et al. [30] (PMID: 23418735)	2013	Case series, monocentric, retrospective, interventional study	Full thickness arcuate (semicircular) retinotomy temporal to a macular hole: at 500–700 μm from the temporal margin of the macular hole, centered 90° on the temporal horizontal meridian	6	Disposable 25G curved scissors	RPE defect at the arcuate retinotomy site	C_3_F_8_ or SF_6_	Five (83%) of six eyes achieved FTMH closure and three (50%) evidenced an improvement in visual acuity Postoperative OCT evaluation: closed retinotomies in all cases
Karacorlu et al. [34] (PMID: 28221260)	2019	Case report	Double (inferior and superior) posterior arcuate 120° relaxing retinotomies, near the inferotemporal and superotemporal vascular arcades	1	Scissors	No postoperative or intraoperative complications	Silicon oil	FTMH closure but restricted functional improvement
Knight et al. [33] (PMID: 30865057)	2021	Case report	Single superonasal (to the macula) relaxing retinotomy	1	Endodiathermy	No postoperative or intraoperative complications	C_3_F_8_	FTMH and nasal retinotomy site completely closed with BCVA improving from 20/200 to 20/100 after secondary repair
Tsipursky et al. [32] (PMID: 37007177)	2021	Multicenter, retrospective, interventional study	Paracentral nasal relaxing retinotomy, at the midpoint between the optic disc and the fovea	13	Endodiathermy with a 25G tapered tip	Small paracentral scotomas, well tolerated by the patients	C_3_F_8_ or SF_6_	12 of 13 eyes (92%) attained anatomical closure after a median follow-up of 1 year 9 out of 13 patients implemented vision: mean visual acuity ameliorated considerably from 1.20 ± 0.15 logMAR to 0.84 ± 0.11 logMAR

Abbreviations: FTMH, full-thickness macular holes; 25G, 25-gauge; BCVA, best-corrected visual acuity; VA, visual acuity; C_3_F_8_, perfluoropropane; SF_6_, sulfur hexafluoride; logMAR, logarithm of minimum angle of resolution; RPE, retinal pigment epithelium. ^a^ First author.

## Data Availability

Data sharing is not applicable.
